# An emerging role for the anti-inflammatory cytokine interleukin-10 in dengue virus infection

**DOI:** 10.1186/1423-0127-20-40

**Published:** 2013-06-25

**Authors:** Tsung-Ting Tsai, Yi-Jui Chuang, Yee-Shin Lin, Shu-Wen Wan, Chia-Ling Chen, Chiou-Feng Lin

**Affiliations:** 1 Institute of Basic Medical Sciences, College of Medicine, National Cheng Kung University, Tainan 701, Taiwan; 2 Institute of Clinical Medicine, College of Medicine, National Cheng Kung University, Tainan 701, Taiwan; 3 Department of Microbiology and Immunology, College of Medicine, National Cheng Kung University, Tainan 701, Taiwan; 4 Center of Infectious Disease and Signaling Research, National Cheng Kung University, Tainan 701, Taiwan

**Keywords:** DENV, Antibody-dependent enhancement, Infection, IL-10, Immunopathogenesis

## Abstract

Infection with dengue virus (DENV) causes both mild dengue fever and severe dengue diseases, such as dengue hemorrhagic fever and dengue shock syndrome. The pathogenic mechanisms for DENV are complicated, involving viral cytotoxicity, immunopathogenesis, autoimmunity, and underlying host diseases. Viral load correlates with disease severity, while the antibody-dependent enhancement of infection largely determines the secondary effects of DENV infection. Epidemiological and experimental studies have revealed an association between the plasma levels of interleukin (IL)-10, which is the master anti-inflammatory cytokine, and disease severity in patients with DENV infection. Based on current knowledge of IL-10-mediated immune regulation during infection, researchers speculate an emerging role for IL-10 in clinical disease prognosis and dengue pathogenesis. However, the regulation of dengue pathogenesis has not been fully elucidated. This review article discusses the regulation and implications of IL-10 in DENV infection. For future strategies against DENV infection, manipulating IL-10 may be an effective antiviral treatment in addition to the development of a safe dengue vaccine.

## Review

### Dengue virus infection

Infection with the four serotypes of dengue virus (DENV), a mosquito-borne virus belonging to the family *Flaviviridae*, causes a global burden of 50 million infections per year occurring across approximately 100 countries [1]. DENV infection results in a wide range of disorders, ranging from mild dengue fever (DF) to severe dengue hemorrhagic fever (DHF) and dengue shock syndrome (DSS), which can cause death in the absence of appropriate medication [1]. Dengue patients frequently present clinical symptoms ranging from a mild fever to an incapacitating high fever with severe headache, pain behind the eyes, muscle and joint pain, and rash. However, in patients with severe DHF/DSS, potentially lethal complications include plasma leakage, severe hemorrhage, and organ failure, and these complications can affect both children and adults [1,2].

The enveloped single-stranded RNA virus of dengue virus (DENV) contains 3 structural proteins, including the envelope (E) protein, the precursor membrane (prM) protein, and the capsid protein, and 7 nonstructural (NS) proteins, including NS1, NS2A, NS2B, NS3, NS4A, NS4B, and NS5 in endoplasmic reticulum (ER)‑derived membrane structures. The newly synthesized viral RNA is incorporated into viral proteins and assembled into immature virions within the ER lumen [3-5]. The life cycle of DENV starts with the entry of infectious virions into target cells through membrane fusion and the binding of surface receptors/co-receptors. Most DENV proteins play a crucial role in the biological functions and pathogenesis of DENV. The DENV E protein is a viral receptor for cell binding and fusion in monocytes/macrophages, dendritic cells, B cells, T cells, basophil/mast cells, endothelial cells, epithelial cells, and hepatocytes [6,7]. Several surface molecules, including heparan sulfate [8], CD14 [9], dendritic cell-specific intracellular adhesion molecule 3 grabbing nonintegrin [10], GRP78 [11], laminin receptor [12], heat shock proteins [13], mannose receptor [14], C-type lectin domain family 5 member A [15], and integrins [16], are required for cell binding and entry during DENV infection. After receptor binding, DENV infects target cells through receptor-mediated endocytosis [7]. When the virions are internalized through endocytosis, the surface E protein is rearranged under environment acidification, leading to viral and vesicle membrane fusion and the subsequent release of viral RNA into the cytoplasm. Cytosolic NS1 and NS2A proteins control viral RNA replication complexes while NS4B modulates DENV replication via interactions with NS3 [17-20]. In addition, both soluble NS1 and membrane-bound NS1 proteins may play a role in complement activation following the binding of anti-NS antibodies [21-23]. The serum levels of soluble NS1 predict DHF progression [24]. The NS2B protein, which is a co-factor of NS3, forms a complex with NS2A/NS3 to regulate viral replication, post-translation modification, and virion assembly through multifaceted enzyme activities, including RNA helicase, RNA 5′-triphosphatase (RTPase), and RNA-stimulated nucleoside triphosphatase (NTPase) activity [25]. The NS5 protein, which is the largest and most highly conserved DENV protein, acts as an RNA-dependent RNA polymerase [26] and methyltransferase [25], and it interacts with the helicase domain of NS3, triggering its RTPase and NTPase activities [27,28] during DENV replication.

Unfortunately, no safe dengue vaccine is available, even though considerable effort has been directed toward the development of several candidate vaccines [29-31]. The biggest challenge is the lack of a clear antiviral strategy, reflecting the multifaceted pathogenesis, including viral load; virulence; cytotoxicity; the nature of the immune response; autoimmunity [32,33]; and the potential effects of underlying host diseases, such as allergies, diabetes, and hypertension [34,35].

### Dengue pathogenesis

The pathogenesis of DENV infection is classified into several types, including viral factors, cytokine storms, host genetic factors, autoimmunity, and antibody-dependent enhancement (ADE) [33,36-39]. Many reports have suggested that the viral genotypic nucleotide variation is associated with disease severity [40,41]. In addition, higher levels of plasma DENV RNA have been observed in DHF patients compared with DF patients [42,43]. More data are needed to conclusively correlate viral load with disease severity. Interferons (IFNs) are central players in the innate immune system for defense against pathogen infection. However, DENV harbors a number of virulence proteins that interfere with the IFN signaling pathway [4,44-48]. The NS2A, NS4A, and NS4B proteins contribute to immune invasion by disrupting type I IFN signaling [44,46,47]. Furthermore, NS5 inhibits IFN-α signaling by inhibiting signal transducer and activator of transcription (STAT) 2 phosphorylation [45].

In addition to viral factors, including viral load, serotype, and virulence, a number of proinflammatory and anti-inflammatory responses are generated in host cells that have been infected with DENV. While aberrant inflammatory responses have been identified in DENV-infected patients, a number of cytokines, including tumor necrosis factor (TNF) α, IFN-γ, granulocyte-macrophage colony-stimulating factor, interleukin (IL)-10, and soluble TNF-α receptors (sTNFR) I and sTNFRII, exhibit greater expression in DHF/DSS patients compared with DF patients [49-54]. The immunopathogenesis of DENV infection involves host-specific immune responses, including immune cell activation, the release of cytokines (IL-1β, IL-2, IL-6, IL-10, IL-13, IL-18, macrophage migration inhibitory factor, tumor growth factor-β, TNF, and IFNs) and chemokines (IL-8, monocyte chemoattractant protein-1, and regulated and normal T cell expressed and secreted), complement activation, the production of inflammatory mediators, and autoimmunity [6,30,32,33,38,55,56]. Recently, based on genome-wide association studies has determined that host genetic factors, including the human leukocyte antigens, antibody receptors, immune/inflammatory mediators, attachment molecules, cytokines, and other immunoregulatory factors, are associated with the pathogenesis of severe dengue [37].

During infection, antibodies against soluble NS1 may lead to the complement-mediated lysis of DENV-infected cells [23]. For DENV-induced autoimmunity, anti-DENV NS1 antibodies bind to human platelets and endothelial cells [57,58]. Numerous studies [6,59-62] have reported mechanisms of molecular mimicry in which antibodies directed against DENV NS1 cross-react with human platelets and endothelial cells and cause damage and dysfunction, which may also be associated with the clinical features of dengue disease. The C-terminus of NS1 may be responsible for cross-reactivity with endothelial cells and platelets, as demonstrated through experiments using a modified NS1 lacking cross-reactive epitopes [63]. In addition, the deletion of the C-terminus of DENV NS1 abolishes anti-NS1-mediated platelet dysfunction and associated bleeding [63]. In addition, antibodies against DENV E and prM proteins also have autoimmune potential. Monoclonal anti-E antibodies bind to coagulant factor, and anti-prM antibodies bind to host cells [64,65]. Autoimmunity might therefore be involved in DENV pathogenesis; however, the timing of autoantibody generation and generated titers associated with clinical parameters need further clarification. Furthermore, the generation of autoantibodies may cause safety concerns for vaccine development.

Humoral immunity is commonly involved in DHF/DSS pathogenesis, particularly in patients with a secondary DENV infection. ADE, a phenomenon in which non-neutralizing antibodies cross-react with heterogeneous serotypes of DENV and facilitate their binding with Fcγ receptor-bearing cells, facilitates severe DHF/DSS during DENV infection [55,66-68]. The generation of antibodies against the DENV E and prM proteins is fundamental for host defense; however, such immune responses may increase the risk of developing DHF/DSS upon re-infection, primarily due to the effects of ADE. In addition to the extrinsic ADE pathway, in which the Fcγ receptor directly facilitates DENV binding onto the cell surface for DENV infection/replication, an intrinsic ADE pathway induces IL-10-mediated immunosuppression [55,69]. For the intrinsic pathway, the ADE of DENV infection triggers IL-10 production through an immune complex associated with the Fcγ receptor to enhance the infection severity. In the presence of ADE, the Fcγ receptor can facilitate viral entry and trigger intracellular signaling. Moreover, IL-10 overproduction can enhance downstream signaling protein suppressor of cytokine signaling (SOCS) 3 expression, followed by type I IFN signaling suppression in the human monocyte cell line THP-1 [69,70]. However, the molecular mechanisms of host and viral regulation of IL-10 expression and the pathological role of IL-10 in DENV infection are mostly unknown. Therefore, the generation of autoimmunity and ADE may cause concerns for vaccine development against DENV infection. Both viral particles acting through the extrinsic pathway and Fcγ receptor signaling through the intrinsic pathway are important for IL-10 induction. To clarify the potential effects of these regulatory routes, determining the detailed molecular mechanisms underlying DENV-induced IL-10 production is an important target for research.

### IL-10 expression and activation

The balance between inflammation and anti-inflammation is critical for infection control [71,72]. IL-10, which was originally named cytokine synthesis inhibitory factor, is a cytokine that is produced by type 2 T-helper cells [73]. IL-10 exhibits anti-inflammatory properties, including the inhibition of immune mediator secretion, antigen presentation, and phagocytosis [74]. Currently, 6 IL-10-related cytokines, including IL-10, IL-19, IL-20, IL-22, IL-24, and IL-26, have been identified [75,76]. All IL-10 family members utilize similar receptor complexes. Two transmembrane glycoproteins, IL-10 receptor (IL-10R) 1 and IL-10R2, form the complete IL-10R. There are 2 steps involved in the initiation of IL-10 signaling. IL-10 first binds to IL-10R1, and the interaction between IL-10/IL-10R1 changes the conformation of the IL-10/IL-10R1 complex to facilitate the interaction between IL-10/IL-10R1 and IL-10R2 [77]. The cross-reaction of IL-10Rs induces the Janus kinase (Jak) 1/Tyrosine kinase (Tyk) 2-mediated phosphorylation of IL-10R1 at tyrosine residue 446/496. Subsequently, STAT3 binding induces autophosphorylation [78,79], followed by downstream gene transcription. A recent study showed that numerous immune cells, including dendritic cells, monocytes/macrophages, B cells, T cells, nature killer (NK) cells, mast cells, neutrophils, and eosinophils, produce IL-10 *in vivo* or *in vitro*[80].

### Regulation of IL-10 production in DENV infection

In DENV-infected cells, a variety of immune mediators alter anti-viral responses and inflammatory activation [6,38]; however, the mechanisms for such responses are in need of further investigation. Increased levels of serum IL-10 may be a useful prognostic hallmark in DHF/DSS patients, as discussed above. Aberrant IL-10 expression may also be involved in DENV pathogenesis, particularly for DENV infection/replication under ADE as demonstrated *in vitro*[55]. However, the significance of this *in vivo* IL-10 expression is not known.

IL-10 is a cytokine with pleiotropic effects in immunoregulation and inflammation. IL-10 may play a role in DENV pathogenesis, reflecting an immunosuppressive function that causes IFN resistance, followed by impaired immune clearance and a persistent infectious effect for acute viral infection. Duell and colleagues [81] summarized IL-10 induction in distinct pathogens. Microbes, including protozoa, nematodes, fungi, viruses, and bacteria, regulate host cell IL-10 expression to allow persistent infection [82-84]. In Table 1, we summarize a panel of epidemiological studies from the past decade that report a positive correlation between IL-10 levels and dengue disease severity [50,85-92]. Overall, higher levels of IL-10 are detected in DHF/DSS patients compared with DF patients, and this trend is observed for infants, children, and adults. The time-kinetic analysis shows increased levels of IL-10 from the onset of fever to defervescence, and viremia primarily occurs during fever in dengue patients [50,85,93]. The relationship between IL-10 and viral replication is therefore speculated, and the possible pathogen effects may result from the IL-10-mediated inhibition of the antiviral IFN response [55]. Another study showed a late peak of IL-10 production after viremia at defervescence [90]. Maximal plasma IL-10 levels measured from the acute phase of infection correlated with the degree of plasma leakage, as determined by the pleural effusion index [50,90]. Thus, IL-10 may cause lymphocyte dysfunction through the suppression of the T cell proliferative response to mitogens, which occurs in dengue patients during the early stages of infection [85,94]. Furthermore, having a decreased number of platelets, called thrombocytopenia, has been associated with the presence of IL-10 [85,90,94]. Interestingly, serum IL-10 levels have been strongly associated with the serum levels of hepatic transaminases AST and ALT [91]. Moreover, the level of IL-10 is higher in secondary DENV-infected patients than in primary DENV-infected patients [95,96]. IL-10 induction is associated with severe DENV infection and is a potential biomarker for acute DENV infection [93,94]. Specifically, IL-10 expression acts as predictive marker of death for DHF patients [86].

**Table 1:** The serum/plasma levels of IL-10 in dengue patients

**Patients (Sample size)**	**Population/Age**	**Year**	**Reference**
DHF (n = 20) > DF (n = 22) > OFI^a^ (n = 19)	Children	1999	[50]
Patient (n = 45) > Healthy (n = 15)	10-82 years old	2001	[85]
DHF^b^ (n = 7) > DHF^c^ (n = 13) > DF (n = 12)	7-79 years old	2002	[86]
DHF (n = 33) > DF (n = 66)	Adults	2002-2003	[87]
Patient^d^ (n = 28) > Healthy (n = 23)	Children	2013	[88]
DHF (n = 17) > DF (n = 21)	Children/Adults	2005-2006	[89]
DHF (n = 29) > DF (n = 12)	Children	1994-1997	[90]
DHF/DSS (n = 86) > Healthy (n = 6)	Infants	1998-2002	[91]
DHF (n = 6) > DF (n = 28)	16-59 years old	2004	[92]

^a^*OFI* Other febrile illnesses; ^b^ Non-survivors; ^c^ Survivors; ^d^ Severe cases.

Several possibilities have been proposed to explain DENV-induced regulation of IL-10. IL-10 is primarily produced by monocytes/macrophages, type 2 T-helper cells, and CD4^+^CD25^+^Foxp3^+^ regulatory T cells, which constitute a suppressive T cell population. An early report showed that increased frequencies of CD4^+^CD25^high^ regulatory T cells are present in dengue patients with acute infection [97]. Based on these findings, the ratios of regulatory/effector T cells are also increased. Furthermore, the activation of this cell population and the generation of IL-10 are normal during infection. Activated regulatory T cells may be one of the IL-10-producing cell populations in circulation. Current studies have shown that cell type specificity and host genetic polymorphisms affect IL-10 production during ADE of DENV infection [98]. Specifically, in monocytes, as previously demonstrated [69,70], IL-10 is induced only in ADE infection, but not in DENV infection alone. However, other Fcγ receptor-bearing cells, including dendritic cells, B cells, mast cells, and NK cells, may also produce IL-10 in an ADE-regulated manner. An analysis of the single nucleotide polymorphisms in the IL-10 promoter region revealed that the homozygous GCC haplotype is associated with an increased level of IL-10 [98]. However, another group showed that the IL-10 (−1082/-819/-592) ACC/ATA haplotype is associated with DHF even though this haplotype results in downregulated IL-10 [92]. Although host cell responses and genetic polymorphisms complicate IL-10 regulation, these studies do not support a strong role for IL-10 in ADE-facilitated DHF/DSS progression.

Aberrant production of IL-10 could be the result of intrinsic regulation by ADE in DENV infection [70]. IL-10 activation followed by SOCS3 expression has been demonstrated during ADE in DENV infection and is also observed in patients with DHF/DSS [69]. Following DENV infection of monocytes, IL-10 expression is induced in a time-dependent manner; notably, ADE significantly facilitates this response. This study was the first report to show that DENV and ADE directly co-regulate IL-10, which is increased in severe DHF/DSS patients. To explain the effects of ADE on IL-10 upregulation, intrinsic signaling through Fcγ receptor-mediated sequential activation of splenic tyrosine kinases mitogen-activated protein kinase (MAPK) and extracellular signal-regulated kinase (ERK) has been suggested [55]. This potential molecular mechanism needs further exploration in the near future, particularly at the level of the transcriptional and translational regulation of IL-10.

Various transcription factors are involved in the production of IL-10 by monocytes/macrophages, including activating transcription factor 1, CCAAT/enhancer binding protein-β, cAMP-responsive element-binding protein (CREB), nuclear factor-κB (NF-κB), pre-B-cell leukemia transcription factor 1, PBX-regulating protein 1, specific protein 1, and MAF [99,100]. Notably, these transcription factors are commonly regulated by MAPKs, including p38 MAPK and ERK. Recent studies [101,102] reported that inhibiting glycogen synthase kinase (GSK)-3, a multi-functional serine/threonine kinase that controls protein synthesis, cell proliferation, division, differentiation, motility, inflammation, and apoptosis, downregulates Toll-like receptor (TLR)-mediated inflammatory responses but increases IL-10 production. We recently showed that GSK-3 regulates inflammatory activation in lipopolysaccharide (LPS)-activated macrophages, partly through inhibiting IL-10 [103]. Mechanistically, GSK-3 negatively regulates CREB, a transcription factor that promotes IL-10 [101,102,104]. We recently showed the mechanisms through which IFN-γ upregulates LPS-induced nitric oxide (NO) biosynthesis in macrophages through GSK-3-mediated IL-10 inhibition [105]. In the presence of TLR signaling, inhibiting GSK-3 can increase the phosphorylation of the transcription factor CREB. CREB activation is positively mediated by protein kinase A (PKA)-, phosphatidylinositol 3-kinase/PKB-, and PKC-mediated phosphorylation [106]. In addition to PKA, PKB, and PKC, CREB is also regulated by GSK-3β, which decreases CREB stability by phosphorylating CREB at Ser129 [107,108]. Both of these kinases act upstream of GSK-3 and inactivate GSK-3 through phosphorylation at serine residues [107,109,110]. Another study showed that the overexpression of IL-10 is mediated by GSK-3 inhibition-induced PKC and ERK activation [84]. In *Leishmania* infection, GSK-3 negatively regulates myeloid cell IL-10 production in a PI3K/PKB/CREB-dependent manner [111]. During ADE of DENV infection, Fcγ receptor may also trigger both ERK and PKC signaling [55]. Therefore, GSK-3 may be inactivated during DENV infection, which might be important for DENV-induced IL-10 production.

### Implications of IL-10 in dengue pathogenesis

Consistent with many human viruses, such as human immunodeficiency virus, hepatitis C virus, and Epstein-Barr virus, DENV infection also induces IL-10 production [69,112-114]. In ADE infections, very early IL-10 overproduction is correlated with the suppression of anti-viral responses, indicating that the timing of IL-10 expression is important for immunosurveillance. Extrinsic ADE infection contributes to a high rate of viral infection in Fcγ receptor-bearing cells, whereas the intrinsic ADE effect via IL-10 suppresses the activation of the IFN-mediated antiviral response. For modulating the immune response, SOCS3 plays a key role downstream of IL-10 signaling [115]. Interactions between IL-10 and IL-10 receptors activate the Jak/STAT pathway, leading to downstream gene transcription that promotes the anti-inflammatory response [80,116-119]. Several reports have shown that IL-10 might suppress the immune response through negatively regulating MyD88 expression in mononuclear cells [116,120]. ADE of DENV infection may be the principal cause of IL-10-mediated immunopathogenesis. Strategies to manipulate IL-10 regulation may facilitate the development of a safe DENV vaccine, perhaps by providing a way to protect against the effects of ADE caused by current candidate vaccines.

IL-10 can block NF-κB activity, and NF-κB is critical for TLR-mediated antiviral IFN responses; pro-inflammatory activation; production of IL-2, IL-12, TNF-α, and IFN-γ; and expression of MHC class II antigens and co-stimulatory molecules [71,121]. In severe DHF/DSS patients, the levels of IL-2, IL-12, and IFN-γ are decreased [122]; however, the mechanisms underlying this decrease are still unknown. IL-10 is released to inhibit the action of antiviral NK cells during the immune response to viral infection [123,124]. This release may prolong viral infection, and inhibiting IL-10 might facilitate the antiviral response. High titers of viremia, caused by the ADE of DENV infection, determine the frequency of DHF/DSS progression [39,94,125]. In addition to the involvement of extrinsic ADE-mediated viral infection, delayed viral clearance mediated through IL-10 immunosuppression may be involved in DENV pathogenesis.

The type II T-helper cell-derived cytokine IL-10 typically attenuates the type I T-helper cell-derived IFN-γ-activated Jak/STAT signaling pathway [80,115,117-119]. IL-10-induced SOCS3 can block the interaction of STAT1 and the IFN-γ receptor to inhibit the activation of IFN-γ. IFN-γ activity is important for preventing DENV-induced mortality, as demonstrated in an experimental murine model [126]. An antiviral axis of IFN-γ/inducible NO synthase/NO-mediated control of viral replication is exhibited in host cells that have been infected with DENV. Consistent with the findings that *Bordetella parapertusis*-induced IL-10 limits host cytoprotective IFN-γ responses [127], aberrant IL-10 production may also be required for IFN-γ resistance during ADE of DENV infection. Notably, the ADE of DENV infection causes aberrant production of IL-10, followed by aberrant SOCS3 expression and IFN resistance [69,70]. An intrinsic pathway involving the Fcγ receptor may facilitate DENV infection/replication following IL-10-mediated blockade of antiviral IFN responses. During microbial infection, the generation of such infectious immune complexes may also cause similar IL-10-mediated immunopathogenesis [55].

Molecular mimicry between DENV proteins and host proteins may cause autoimmunopathogenesis in DENV infection [33]. However, the mechanisms through which B cells are activated and immunotolerance is compromised remain unclear. IL-10 can enhance B cell survival, proliferation, maturation, and antibody production [128,129], implying a possible role of IL-10 for autoimmunity during ADE of DENV infection. However, it is still controversial whether IL-10 attenuates autoimmunity by blocking IFN-mediated autoimmune-associated inflammation in lupus [130]. The implication of IL-10 in DENV-induced autoimmunity needs further study.

## Conclusions

**Figure 1:**
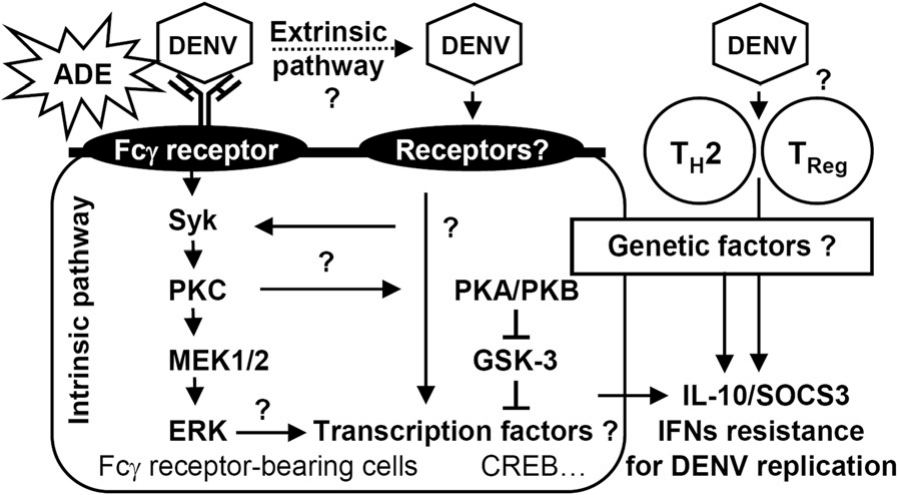
**Theoretical model for IL-10 production and its pathogenic role during DENV infection.** IL-10 is produced in T cells (T_H_2 and Treg) through an unknown mechanism and in Fcγ receptor-bearing cells directly through viral receptors and indirectly through an ADE-facilitated manner. The extrinsic ADE pathway occurs through Fcγ receptor-facilitated virus contact and entry following viral receptor-mediated endocytosis. An alternative intrinsic ADE pathway may trigger Fcγ receptor-mediated signaling to activate Syk/PKC/ERK-regulated IL-10 expression. However, the molecular mechanisms for IL-10 production remain unclear. Crosstalk between PKC/PKA/PKB-regulated GSK-3 for the transcriptional regulation of IL-10 and the involvement of genetic factors are also summarized. IL-10-regulated SOCS3 expression may benefit DENV replication by facilitating IFN resistance.

IL-10 has immunomodulatory effects and is generally considered anti-inflammatory. Excessive or poorly timed IL-10 production may allow viruses to escape from immune surveillance during DENV pathogenesis. DENV-induced IL-10 production, which may be exacerbated by ADE through Fcγ receptor-mediated extrinsic and intrinsic pathways, leads to IL-10/SOCS3-mediated immunosuppression and enhanced viral replication (Figure 1). The molecular basis for IL-10 induction should be investigated in cells during DENV infection and during the ADE of DENV infection. After DENV infection, the major IL-10-producing cells in the host should be identified, and the pathogenic roles of IL-10 must be clarified. In addition, the involvement of viral receptor- and Fcγ receptor-mediated signaling is key for exploring the regulation of IL-10. Targeting IL-10 regulation and signaling pharmacologically using neutralizing antibodies, antagonists, and inhibitors may represent a viable therapeutic strategy for combating the progression of severe dengue diseases.

## Abbreviations

ADE: Antibody-dependent enhancement; CREB: cAMP-responsive element-binding protein; DF: Dengue fever; DHF: Dengue hemorrhagic fever; DSS: Dengue shock syndrome; DENV: Dengue virus; E: Envelope protein; ERK: Extracellular signal-regulated kinase; GSK: Glycogen synthase kinase; GM-CSF: Granulocyte-macrophage colony-stimulating factor; IFN: Interferon; IL: Interleukin; LPS: Lipopolysaccharide; MAPK: Mitogen-activated protein kinase; NO: Nitric oxide; NS: Nonstructural; NF-κB: Nuclear factor-κB; NTPase: Nucleoside triphosphatases; prM: Precursor membrane; PKA: Protein kinase A; RTPase: RNA 5′-triphosphatase; STAT: Signal transducer and activator of transcription; sTNFR: Soluble TNF-α receptor; SOCS: Suppressor of cytokine signaling; TLR: Toll-like receptor; TNF: Tumor necrosis factor.

## Competing interests

The authors declare that they have no competing interests.

## Authors’ contributions

TTT, YJC, YSL, SWW, CLC, and CFL designed the concept, collected information, and prepared the manuscript and figures. TTT, YJC, and CFL wrote the manuscript. All authors read and approved the final manuscript.
